# An educational intervention for improving knowledge of Syrian school children about avulsion using the "save your tooth" poster

**DOI:** 10.1186/s12903-020-01380-4

**Published:** 2021-01-07

**Authors:** Nancy Al Zaher, Mayssoon Dashash

**Affiliations:** 1 Paediatric Dentistry Department, Faculty of Dentistry, Damascus University, Damascus, Syria; 2 Centre for Measurement and Evaluation in Higher Education, Ministry of Higher Education, Damascus, Syria

**Keywords:** Knowledge, Avulsion, Scientific poster, Management

## Abstract

**Background:**

The prognosis of replantation of an avulsed tooth is affected by the first aid management in the first 15 min after traumatic incident. Knowledge of the optimal management is crucial to successful replantation. The objective of this study was to investigate the effectiveness of educational intervention using the Arabic version of the "save your tooth" poster designed by the International Association of Dental Traumatology IADT, in improving the knowledge of schoolchildren about first aid management of avulsion of permanent teeth.

**Methods:**

An interventional educational study was undertaken. A total of 550 schoolchildren aged 9–12 years participated in this study. Thirteen public primary schools in Damascus city were selected. A questionnaire was developed to measure the knowledge of schoolchildren about avulsion of permanent teeth. The translated IADT education poster about avulsion management was adopted.The content of this poster was explained to the children. Two months later, the subjects were re-evaluated using the same questionnaire. Paired sample *t*-test was used to test the differences existed between the two assessments.

**Result:**

A total of 537 schoolchildren completed the questionnaires in which (n = 305) 57% were females and (n = 232) 43% were males. The findings demonstrated significant improvement in the participants’ responses after interventional education (*P* < 0.05). The mean score of knowledge increased significantly from 3.71 at the baseline to 4.03 after the intervention (*P* < 0.003).

**Conclusion:**

The findings of the present study showed that the level of knowledge of Syrian schoolchildren regarding first-aid management of avulsion of permanent teeth was limited at the baseline. The follow-up results showed that the educational intervention based on the “save your tooth” poster was significantly effective in improving the knowledge of schoolchildren. Further interventions to educate all Syrian schoolchildren about avulsion could be of great value to prevent its negative aesthetic, functional, psychological, economic impacts.

## Background

Traumatic dental injuries (TDIs) can cause several functional, emotional and economic problems. They can cause pain and negatively affect occlusion, as well as dental aesthetics. Moreover, these injuries have a negative impact on the quality of life of children [1, 2]. Previous studies have indicated that 25% of schoolchildren experienced dental trauma [3] and approximately 19% of dental-related injuries will occur at schools [4].

Avulsion, which is a complete displacement of a tooth from its socket in alveolar bone, has been the most critical type of TDIs due to the complex injuries that can cause to periodontal and pulpal tissue [5–7]. It is seen in 0.5–16% of all TDIs [8, 9]. The prognosis of an avulsed tooth depends on appropriate management at the place of accident and the immediate replantation of the avulsed tooth [10, 11] in which the periodontal ligament fibers and the neuromuscular bundles at the root apex can be maintained [12].

Therefore, children, parents, teachers and school nurses should be adequately trained in order to provide first aid at the scene of accident, before attending dental clinics to avoid future consequences that can affect the quality of life of the patient.

Researchers have reported a low level of knowledge about first aid management of dental trauma including avulsion [13–15]. For instance, Adekoya-Sofowora et al. reported low level of knowledge regarding tooth avulsion and indicated that cultural belief of throwing a knock out or exfoliated tooth on the roof had negative impact on children [8]. Similarly, Andersson et al. reported low level of knowledge about tooth avulsion and replantation, extra-alveolar time and storage media among Kuwaiti schoolchildren [16].

Previous studies were undertaken to improve knowledge about dental trauma among societies through implementing public awareness campaigns, distributing leaflets, posters, and educational presentations. Researchers have demonstrated the effectiveness of these activities and interventions in preventing dental trauma and providing first aid management of avulsion [3, 17–20].

In Syria, the number of patients attending clinics with TDIs has increased during the Syrian crisis with no data to show the exact prevalence [21]. Researchers have suggested implementation of effective continuous education in TDIs during the crisis to fulfill the responsibility of the current situation [21]. No reported Syrian study assessed the level of knowledge among Syrian children about avulsion and replantation. Therefore, this study was undertaken to assess the level of knowledge among Syrian schoolchildren and investigate the effectiveness of educational interventions in improving the knowledge and awareness of Syrian schoolchildren in Damascus about avulsion in permanent teeth.

## Materials and methods

### Study design

An interventional educational study was undertaken in the city of Damascus, Syria, between September 2018 and November 2018.

The list published by the Syrian Ministry of Education, which contained 225 primary schools in Damascus was adopted. The city of Damascus was divided into 2 areas; the first area (Area 1) which contains 108 primary schools, represents people with high educational and social status and Area 2, which contains 117 primary school, related to people with lower status. Thirteen elementary schools, (six primary schools in Area 1 and seven from Area 2) were randomly selected from the list obtained from the Ministry of Education. Schoolchildren aged 9–12 year old were invited for participation.

### Sample

The minimum sample size to satisfy the requirement was estimated to be 537 to achieve a level of precision with a standard error of 5%. The required sample size was increased to 550 to avoid Type II sampling error, decrease the effect of confounding variables and increase the precision of the study [22]. Loss to follow-up, missing data and withdrawals from the experiment were also considered. The initial sample size was 240 boys and 310 girls. The number of participants decreased to 537 (232 boys and 305girls) during the recall visits due to children having moved to other schools or being absent the day of post intervention.

### Ethical approval

Ethical Approval was obtained from the Research and Ethical Committee of the Faculty of Dentistry in Damascus University, Syria (No. 543/4408) dated 12–9-2018. In addition, a formal permission was obtained from the Ministry of Education in order to get access to schools and perform the required examinations on children. Informed consent was obtained from the parents of all participants after explaining the purpose of the study.

### Educational tools

#### Poster and questionnaire design

The current study utilized the Arabic IADT poster “save your tooth”, which was previously translated by one of the authors of this study (MD) for the International Association of Dental Traumatology IADT (https://www.iadt-dentaltrauma.org/for-patients.html) [23], in order to assess the knowledge of Syrian schoolchildren about avulsion.

Besides the poster, the assessment was performed using a dual‑part questionnaire: the first part contained the participants’ demographic information (age, sex, name of school), and the second part was related to the awareness, knowledge, and attitude towards dental trauma emergency protocol for tooth avulsion (Additional file 1).

The questionnaire was pretested in a pilot study of 25 randomly selected children who were not part of the main study. Children were asked to complete the questionnaire on two different occasions. High concurrence between the answers to the questions on both occasions was observed. Cronbach’s α was used to test the internal consistency of the questionnaire [24] which was found to be 0.83.

#### Educational intervention

The educational intervention included presenting the content of the Arabic version of the IADT “save your tooth” poster to children by the principal investigator (NAZ) as a short story, explaining the methods of providing first aid management of avulsion, answering questions, and making sure that children understand the poster and contents. The evaluation was undertaken immediately before, and after 8 weeks of the intervention using an identical questionnaire to measure the differences.

### Statistical analysis

Data from the questionnaires were analyzed using SPSS version 25. Descriptive statistical analysis was carried out. The mean score of knowledge for each participant was collected during the two study times. Paired sample t-test was used to test the differences existed between the two assessments. The Chi square test was used for evaluating the changes of the percentage of correct answers after the intervention. Level of significance and confidence interval were set at 5 and 95%, respectively. *P* value less than 0.05 was considered statistically significant.

## Results

A total of 537out of 550 schoolchildren completed the questionnaire. The response rate was 97%. There were 305 (57%) females and 232 (43%) males. There were 244 (45%) children related to Area 1 and 293(55%) living in Area 2. Demographic characteristics of the study sample are presented in Table 1.

**Table 1:** Demographic characteristics of study sample

Sex		Age	Area (%)		
n (%)		Mean ± SD	Area 1	Area 2	Total
Male	232 (43)	10.56 ± 0.97	107 (44)	125 (43)	232 (43)
Female	305 (57)	10.57 ± 0.98	137 (56)	168 (57)	305 (57)
Total	537 (100)	10.56 ± 0.97	244 (45)	293 (55)	537 (100)

The frequencies and percentages of answers reported by the participants before and after educational intervention are presented in Table 2. The findings show that the participants’ responses improved significantly after the intervention (*P* < 0.05). For instance, the number of children who reported that they would look for the tooth directly when avulsion occurs increased significantly from 209 (38.9%) at the baseline, to 475 (88.5%) after 8 weeks (*P* = 0.000). In addition, the number of children who indicated that they would hold the tooth by the crown also increased significantly (*P* = 0.000) from 448 (83.4%) at the baseline to 485 (90.3%) after the educational intervention (*P* = 0.000). Moreover, the number of children who reported that they would save the tooth in milk until replantation significantly improved from 89 (16.6%) before the educational session to 401 (74.7%) after the intervention (*P* = 0.000). The number of children who would seek professional help for an avulsed tooth also significantly improved from 393 (74.3%) to 508 (94.6%) between the two visits, (*P* = 0.000). The paired sample *t*-test indicated that the mean score of knowledge increased significantly from 3.71 at the baseline to 4.03 after educational intervention. Findings are presented in Table 3.

**Table 2:** The frequencies and percentages of answers reported by the participants before and after educational intervention

Questions	Pre		Post		*P* value*
	Correct answer n (%)	Incorrect answer n (%)	Correct answer n (%)	Incorrect answer n (%)	
Q: What is the first thing you would do when avulsion of a tooth occurs? Answer: Look for the tooth	209 (38.9)	328 (61.1)	475 (88.5)	62 (11.5)	0.000
Q: Where would you hold the tooth? Answer: From the crown	448 (83.4)	89 (16.6)	485 (90.3)	52 (9.7)	0.000
Q: Would you clean the tooth? Answer: Yes	465 (86.6)	72 (13.4)	491 (91.4)	46 (8.6)	0.001
Q: If you would clean the tooth, how would you do that? Answer: Rinse in cold tap water	305 (56.8)	232 (43.2)	331 (61.6)	206 (38.4)	0.02
Q: How would you store the tooth until you visit a dentist? Answer: Milk	89 (16.6)	448 (83.4)	401 (74.7)	136 (25.3)	0.000
Q: If you don’t find a storage media to store the tooth, where would you put the tooth? Answer: In the mouth between cheeks and gums	78 (14.5)	459 (85.5)	449 (83.6)	88 (16.4)	0.000
Q: When should you go to a dentist? Answer: Immediately,within the first 30 min from the injury	393 (74.3)	138 (25.7)	508 (94.6)	29 (5.4)	0.000

*P values derived from Chi square test

**Table 3:** comparison of the mean score of knowledge between the two visits

	Mean score of knowledge ± SD		
	Baseline	Post 8 weeks	*P* value
Mean score of knowledge	3.71 ± 1.26	4.03 ± 2.19	*t* = 2.935 *P* < 0.003*

*Paired sample *t-test*

## Discussion

Several studies were undertaken to assess the knowledge of teachers, parents, and children about the management of TDIs and indicated inadequate awareness [18, 25–31]. In the case of an avulsed permanent tooth, the most critical aspect that affects the long-term prognosis of the tooth is the length of time the tooth stays out of its socket [3].

Clinical studies have demonstrated that the immediate appropriate replantation as well as the use of proper storage medium are able to regenerate periodontal ligament of the avulsed tooth after replantation [16]. Moreover, they demonstrated that the vitality of the periodontal ligament cells would not be sustained if the extra oral dry time were longer than 60 min [3]. Therefore, there is a critical need to deliver a simple message to children, teachers, nurses and parents that the best course of action to save the avulsed permanent tooth, is to immediately and appropriately place it back in its socket [10].

Previous studies have employed various versions of the IADT poster “save your tooth” to improve the knowledge of children, teachers, nurses and parents about avulsion of permanent teeth [3, 32]. The Arabic IADT poster “save your tooth” is easily accessible and available free to public. It was found to be an effective educational tool, easy to understand and to implement [32]. The current study utilized the Arabic IADT poster “save your tooth” which was previously translated by one of the authors of this study (MD) for the IADT (https://www.iadt-dentaltrauma.org/for-patients.html) [23] in order to assess the knowledge of Syrian schoolchildren about avulsion.

To our knowledge, this study is the first to investigate the effectiveness of educational intervention in improving the knowledge of Syrian schoolchildren about avulsion in permanent teeth. Previous work has demonstrated an increased number of cases of dental traumatic injuries attending dental clinics during the Syrian crisis with no or limited data estimating the exact prevalence of the problem [21].

The questionnaire administered in this study was carefully planned to enable and facilitate the evaluation of knowledge gained through education. Findings showed that the level of knowledge of Syrian primary schoolchildren regarding first-aid management of avulsion was inadequate at the baseline. The lack of knowledge could possibly mean that the avulsed tooth might not be replanted at all, or not receive the optimal urgent management in which a poor prognosis might occur. This is disappointing because the outcome would be very different if simple procedures were applied [16]. Similar findings were reported by Nigerian researchers who demonstrated low level of knowledge among school children about avulsion and reported that many Nigerian children would not replant an avulsed tooth [8].

The present findings were also similar to those reported by a Kuwaiti study [33], which suggested increasing the knowledge about management of avulsion in the society in order to prevent future negative consequences and high cost of replacing a permanent tooth for the individual, the family and the society [8, 33]. Findings of follow-up showed that the intervention was effective in improving the knowledge of schoolchildren. There was a positive and statistically significant change in awareness from baseline to 8 weeks in all questions investigated. For example, the number of participants who preferred milk for transferring an avulsed tooth increased from 16.6 to 74.7% after the intervention.

Bistrickienė (2019) reported an improvement in the knowledge regarding tooth transport media after an educational intervention. The number of respondents who knew it would be safe to keep the avulsed tooth in milk increasd from 5.52 to 54.26% and the number of children who selected saliva increasd also from 6.21 to 44.68% after the intervention [34].

The IADT guidelines [10] recommended rinsing the avulsed tooth for about 10 s under running water for a dirty avulsed tooth. A significant improvement in the knowledge among study participants regarding rinsing the avulsed tooth was observed after intervention (*P* = 0.02). Similarly, AL Sari and co-workers (2019) reported improvement in the knowledge regarding how to clean an avulsed tooth when they used the same poster to improve knowledge of nurses and physical education teachers [3]. Additionally, improvement of knowledge was noted after the intervention (*P* = 0.000) as children selected the correct answer regarding seeking help within 30 min of an avulsed permanent tooth. Similar improvement was observed in Bistrickienė’ s study (2019) who reported an increase in the number of children from only 7.8 to 90.3% who kept the lost tooth for no longer than 30 min as a safe time until replantation [34]. The useful information delivered in an easy and entertaining way together with the health messages presented as a short story with colorful images might be the elements that led to improvement. Al Bardaweel and Dashash (2018) reported that educational posters that include information and pictures of the subject might be an effective material in improving knowledge for a topic such as first aid injury management [35].

Further research is suggested to investigate whether the interaction with the dentist, who delivered the education, using the poster, is bound to have positive influence on the children and can improve their health practice.

That educational intervention significantly improved knowledge on the emergency management of (TDI) among primary schoolchildren. Castilho et al. provided evidence that the educational campaigns for prevention of accidents involving the dental tissues and for improvement of the prognosis of avulsed teeth are noteworthy as an urgent necessity [36].

This study provides valuable insight into the importance of undertaking educational intervention to improve the knowledge of children about avulsion. The Arabic IADT poster has been an effective simple tool that can be utilized for improving knowledge about avulsion of permanent teeth. Similar intervention programs should be undertaken to improve knowledge of all Syrian schoolchildren.

## Conclusion

The findings of this study have addressed the importance of educational intervention in improving the knowledge of schoolchildren about avulsion. Further interventions are essential to educate all Syrian schoolchildren and society about avulsion of permanent teeth and prevent its negative aesthetic, functional, psychological, and economic impacts.

## Supplementary information

**Additional file 1.** Questionnaire for Assessing Knowledge of kids about saving knocked out tooth.

## Data Availability

The datasets used and/or analyzed during the present study are available from the corresponding author upon a reasonable request.

## Abbreviations

TDI: Traumatic Dental Injury
IADT: International Association of Dental Traumatology
MD: Mayssoon Dashash
NAZ: Nancy Al Zaher
SD: Standard deviation
