# Supplementary material for: An educational intervention for improving knowledge of Syrian school children about avulsion using the "save your tooth" poster
Source: BMC Oral Health. 2021 Jan 7;21:24. doi: 10.1186/s12903-020-01380-4 (PMC7792023; doi:10.1186/s12903-020-01380-4)
Supplement: Supplementary file 1 — Additional file 1. Questionnaire for Assessing Knowledge of kids about saving knocked out tooth. [file 12903_2020_1380_MOESM1_ESM.docx]

| **Questionnaire for Assessing Knowledge of kids about saving knocked out tooth**  **Name: Age: Gender: School Name:**  **Hello my friends… Can you help me answering the following?**  **Select one answer for each of the following questions:**  1-What is the first thing you would do when you hit your front tooth and it knocked out completely?   - Look for the tooth, and save it - stop the bleeding by pressing the hole of the gum without looking for the tooth.   2- How would you hold the tooth?   - From the crown (white part) - From the root (brown part)   3- Would you clean the tooth?   - Yes - No   4- If you would clean the tooth, how would you do that?   - Rinse in cold tub water - Normal saline - Alcohol - Others   5- How would you save the tooth before seeing a dentist?   - Put the tooth in water - Put the tooth in handkerchief - Put the tooth in milk   6- If you don‘t find a container and a solution to store the tooth, where would you put the tooth?   - Put the tooth in the mouth between cheeks and gums - Put the tooth in the hand or pocket   7- When should you go to a dentist?   - Immediately, within the first 30 minutes from the injury - Later, in the next day |
| --- |
